# Renal denervation with standard radiofrequency ablation catheter is effective in 24-hour ambulatory blood pressure reduction – follow-up at 1/3/6/12 months

**DOI:** 10.1007/s12471-016-0839-1

**Published:** 2016-05-10

**Authors:** D. Prochnau, S. Otto, H-R. Figulla, R. Surber

**Affiliations:** Department of Internal Medicine I, Jena University Hospital, Jena, Germany

**Keywords:** Resistant hypertension therapy, Radiofrequency, Renal denervation, 24‑hour ambulatory blood pressure, Standard electrophysiology catheter

## Abstract

**Aims:**

To examine the effect of renal denervation (RDN) on 24‑h ambulatory blood pressure (ABP) with a standard radiofrequency ablation catheter (RF catheter).

**Methods:**

Seventy-five patients with resistant hypertension received bilateral RDN with an RF catheter (6 RF applications, 1 minute each, 8–12 watts). Seventy patients fulfilled inclusion criteria with mean systolic ABP ≥140 mmHg (mean 165/89) despite treatment with ≥3 antihypertensive drugs (mean 5.9) including a diuretic, and were further analysed for ABP changes. Follow-up at 1/3/6/12 months comprised biochemical evaluations and ABP measurement. At 6/12 months, duplex sonography of the renal arteries was additionally performed.

**Results:**

At 1/3/6/12 months we observed a significant reduction in systolic ABP of −15/−17/−18/−15 mmHg (*n* = 55/53/57/50; non-parametric Friedman test, *p* < 0.001) and diastolic ABP of −6/−9/−10/−7 mmHg (*p* < 0.001). Of the patients, 70 %/64 % showed a systolic ABP reduction of ≥10 mmHg, and 77 %/70 % of ≥5 mmHg at 6/12-month follow-up. Two patients (2.7 %) developed renal artery stenosis (>70 %) with subsequent stenting without complications. Logistic regression analysis with systolic ABP reduction ≥10 mmHg at 12 months follow-up as criterion revealed that only the mean baseline systolic ABP was significant, OR = 2.174.

**Conclusions:**

RDN with a standard RF catheter can be used safely to reduce mean ABP in resistant hypertension as shown in long-term follow-up.

## Introduction

Arterial hypertension is a major global health problem that affects 30–45 % of the general population [1]. Cardiovascular complications are common in hypertensive patients. Thus, it is mandatory to achieve a fast and adequate blood pressure (BP) control. The first step in the antihypertensive treatment should always be lifestyle modification followed by an appropriate medical therapy [2], including the use of single-pill combinations and the avoidance of interfering substances [3]. However, an increasing number of especially elderly patients do not respond to antihypertensive therapy in an adequate manner [4]. Resistant hypertension is diagnosed when BP levels remain above 140/90 mmHg despite the use of three antihypertensive drugs at optimal dosages, including a diuretic [1]. As a new invasive treatment for resistant hypertension, the successful use of catheter-based renal denervation (RDN) was first described in the two landmark studies HTN-1 and HTN-2 [5, 6]. In both studies significant reduction of office-based BP following bilateral RDN was demonstrated with an on-going treatment effect up to three years of follow-up [7, 8]. In 2014 the first blinded randomised controlled RDN trial with the large number of 535 patients was published. In contrast to the HTN-1 and HTN-2 trials it did not show differences in BP lowering between RDN and a sham-control procedure [9]. This study has raised important issues, especially by providing evidence for the existence of a placebo effect and raising suspicion for improvement of medication adherence among study patients [9]. However, despite the major impact of this large randomised sham-controlled trial, it had some important methodological issues. More specifically, some of the operators were inexperienced in the field of RDN (with only three or fewer RDN procedures). Further, the study population comprised 25 % Afro-Americans who are known to respond better to sham procedures than to RDN. Finally, the antihypertensive drug treatment of the patients was not truly stable [10].

To answer the question whether RDN is effective or not in BP lowering in patients with truly resistant hypertension, more well-conducted studies with longer follow-up are needed. For this purpose, in comparison with office-based BP, the 24‑h ambulatory BP (ABP) measurement is a more objective method to identify resistant hypertensive patients. In addition, the 24‑h ABP is a valuable predictor of adverse outcome in hypertensive patients [11].

In this paper, we present 1/3/6/12 month follow-up data of an observational, non-controlled study (before – after single group study) by using a standard radiofrequency (RF) ablation catheter for RDN. In order to treat only truly resistant patients, we consequently measured the 24‑h ABP for a selection of eligible patients and also to evaluate the BP changes during follow-up.

## Methods

### Patient sample

Patients were eligible if they had a long history of resistant hypertension (>6 months) with a stable antihypertensive treatment for at least 4 weeks, were aged >18 years, and were not pregnant. To be included in the analysis, patients had to show a mean baseline systolic 24‑h ABP ≥140 mmHg despite medical treatment with at least three antihypertensive drugs in adequate dosages, including a diuretic. Before enrolment, secondary causes of arterial hypertension, including obstructive sleep apnoea (OSAS), primary hyperaldosteronism, renal artery stenosis (>50 % lumen diameter reduction), pheochromocytoma, Cushing’s syndrome, and aortic isthmus stenosis, had to be excluded. Efficacy endpoint was the mean change in 24‑h ABP. Patients were classified as responders if the mean systolic reduction in 24‑h ABP was ≥10 mmHg. Safety endpoints were all adverse events (acute and during follow-up). The study was performed in concordance with the Declaration of Helsinki. All patients provided written informed consent prior to the intervention.

### Study procedure

Baseline evaluation of patients comprised clinical history, review of medication, physical examination, blood chemistry (including serum creatinine and proteinuria), and 24‑h ABP measurement. The devices took a reading at least every 30 minutes during the day and every 45 minutes during the night. A minimum of at least 14 successful measurements per day and 7 successful measurements per night were considered sufficient. Our RDN procedure has previously been described in detail [12]. In short, renal artery stenosis was excluded by renal angiogram via femoral access using a 5‑French (F) Judkins right 4 catheter (JR4). After this, a 7‑F 4‑mm tip standard steerable RF ablation catheter (Marinr®; Medtronic Inc., Minneapolis, MN, USA) was introduced into the renal artery without using a guiding sheet. RF ablation was performed in both renal arteries, consecutively. We applied six low-power RF applications (1 minute each, 8–12 watts) along the length of the renal arteries, separated both longitudinally and rotationally to achieve a circumferential lesion. During ablation patients received intravenous narcotic and sedative drugs (midazolam and fentanyl), and intravenous unfractionated heparin with an activated clotting time between 250 and 300 seconds. Aspirin (100 mg per day) was given during the following three months.

Follow-up assessment at 1/3/6/12 months consisted of 24‑h ABP measurement, physical examination, blood chemistry (including serum creatinine and proteinuria) and adverse events. Duplex sonography of the renal artery was performed at baseline and at 3 or 6 months and 12 months of follow-up.

### Statistical analysis

Continuous variables are expressed as mean ± standard deviation (SD). Categorical data are summarised as frequencies and percentages. Test results are reported as follows: the test statistic (χ^2^) value, degrees of freedom (df) and the significance level. Subsequent single comparisons between baseline and 1/3/6/12-month follow-up were conducted by Wilcoxon signed-rank tests, utilising a Bonferroni correction for repeated testing (for a nominal 0.05 level the actual significance level was set at *p* < 0.01). To adjust for potential confounding factors in RDN therapy, a multivariate logistic regression model was performed. Odds ratios (ORs) with 95 % confidence intervals (CIs) were calculated as an estimate of mean systolic 24‑h ABP reduction ≥10 mmHg. The statistical analyses were computed with SPSS© (SPSS Inc., Chicago, IL, USA) statistical software.

## Results

### Study population

We performed RDN in 75 patients with resistant hypertension. In one patient the ablation catheter could only be introduced in the left renal artery. In all other patients we successfully performed bilateral RDN. Five patients admitted to our hospital for RDN with a documented home- and office-based systolic BP of >160 mmHg did not show a mean systolic 24‑h ABP ≥140 mmHg and were therefore excluded from our 24‑h ABP analysis. Nevertheless, for safety reasons the follow-up assessment was performed regularly in these five patients. In our 24‑h ABP analysis we included 70 patients (mean age 64 years; 46 male) with severe drug-resistant hypertension (mean baseline 24‑h ABP 165/89 mmHg) despite treatment with at least three antihypertensive drugs (mean 5.9) in optimal doses, including a diuretic (except one patient with end-stage kidney disease). All of the patients insisted they were adherent to the prescribed antihypertensive drug treatment. Baseline parameters of the patients are shown in Tab. 1.

**Tab. 1:** Baseline patient characteristics

Parameter	Mean ± SD or *n* (%)
Male sex	46 (61)
Age (years)	63.7 ± 12
eGFR, (ml/min per 1.73 m^2^)	69.4 ± 24
Fluoroscopy time	6.4 ± 3.5
Comorbidity	
Diabetes mellitus	36 (48)
CAD	18 (24)
Chronic renal insufficiency (creatinine ≥130 µmol/l)	12 (16)
Obstructive sleep apnoea (treated)	14 (19)
Body mass index (kg/m^2^)	31.1 ± 5.5
Number of antihypertensive drugs	5.9 ± 1.4
Beta-blocker	60 (80)
ACE-I/ARB	72 (96)
Aldosterone antagonists	18 (23)
Diuretics	74 (98)
Calcium-channel blockers	58 (77)
Vasodilatators	19 (25)
Alpha-1 blockers	44 (59)
Centrally acting sympatholytics	50 (68)

*CAD* coronary artery disease, *eGFR* estimated glomerular filtration rate, *ACE-I* angiotensin-converting enzyme inhibitor, *ARB* angiotensin receptor blocker

**Tab. 2:** Mean systolic and diastolic BP values at baseline, 1, 3, 6 and 12 months

	Systolic ABP ± SD (mmHg)	Diastolic ABP ± SD (mmHg)	Patients (n)
Baseline	165 ± 21	89 ± 15	70
1 month	149 ± 15	81 ± 12	55
3 months	145 ± 17	81 ± 13	53
6 months	146 ± 18	79 ± 12	57
12 months	146 ± 16	79 ± 13	50

*ABP* 24‑h ambulatory blood pressure; *n* number of patients

### 24‑h ABP changes and predictors of successful BP reduction following RDN

**Fig. 1:**
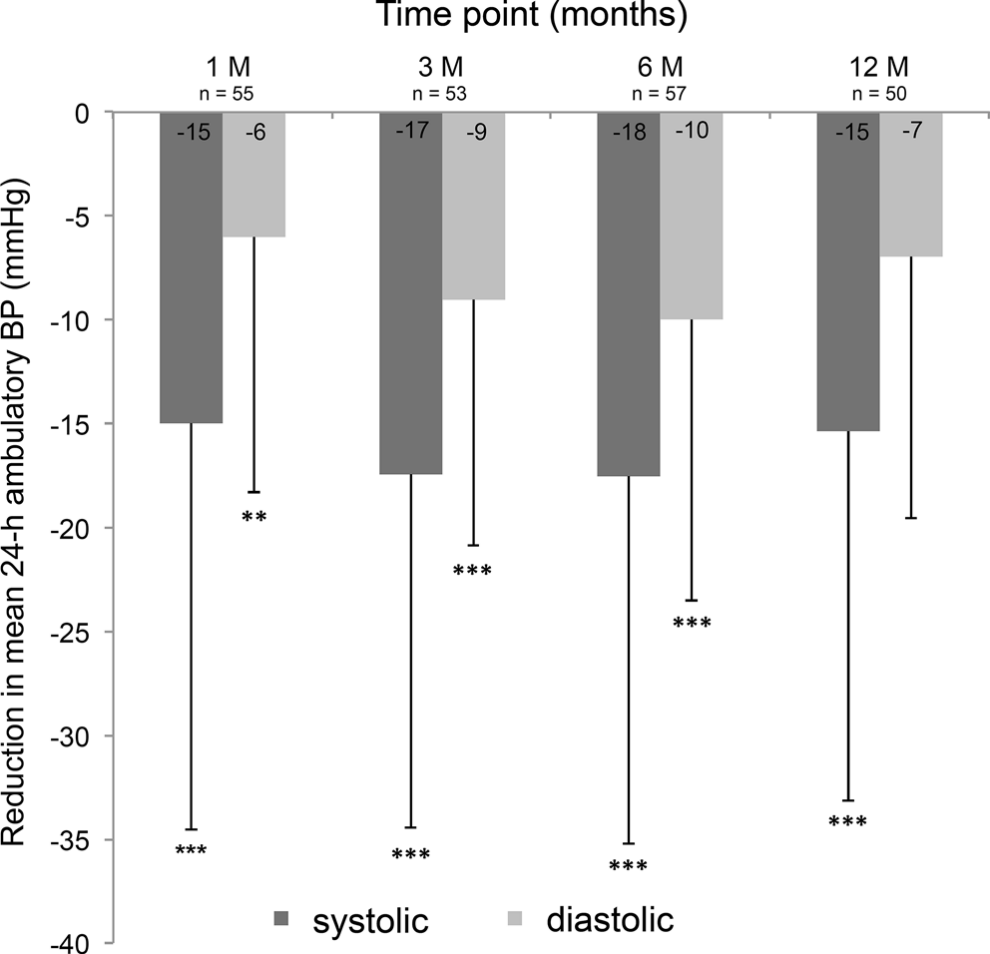
Mean systolic and diastolic blood pressure (BP) changes in the 24‑h ambulatory blood pressure (ABP) following renal denervation at 1/3/6/12-month follow-up. We found a statistically significant reduction in BP following renal denervation with RF in systolic 24‑h ABP (χ^2^ (4) = 26.191, *p* < 0.001) as well as in diastolic 24‑h ABP (χ^2^ (4) = 20.771, *p* < 0.001). Post hoc Wilcoxon signed-rank tests (with Bonferroni correction) showed significant BP changes in systolic 24‑h ABP at 1/3/6/12 months (all *p*‑values < 0.0001) and diastolic 24‑h ABP at 1/3/6 months (all *p*‑values < 0.001) but not at 12 months (*p* = 0.013). (*** *p* < 0.0001, ** *p* < 0.001)

**Fig. 2:**
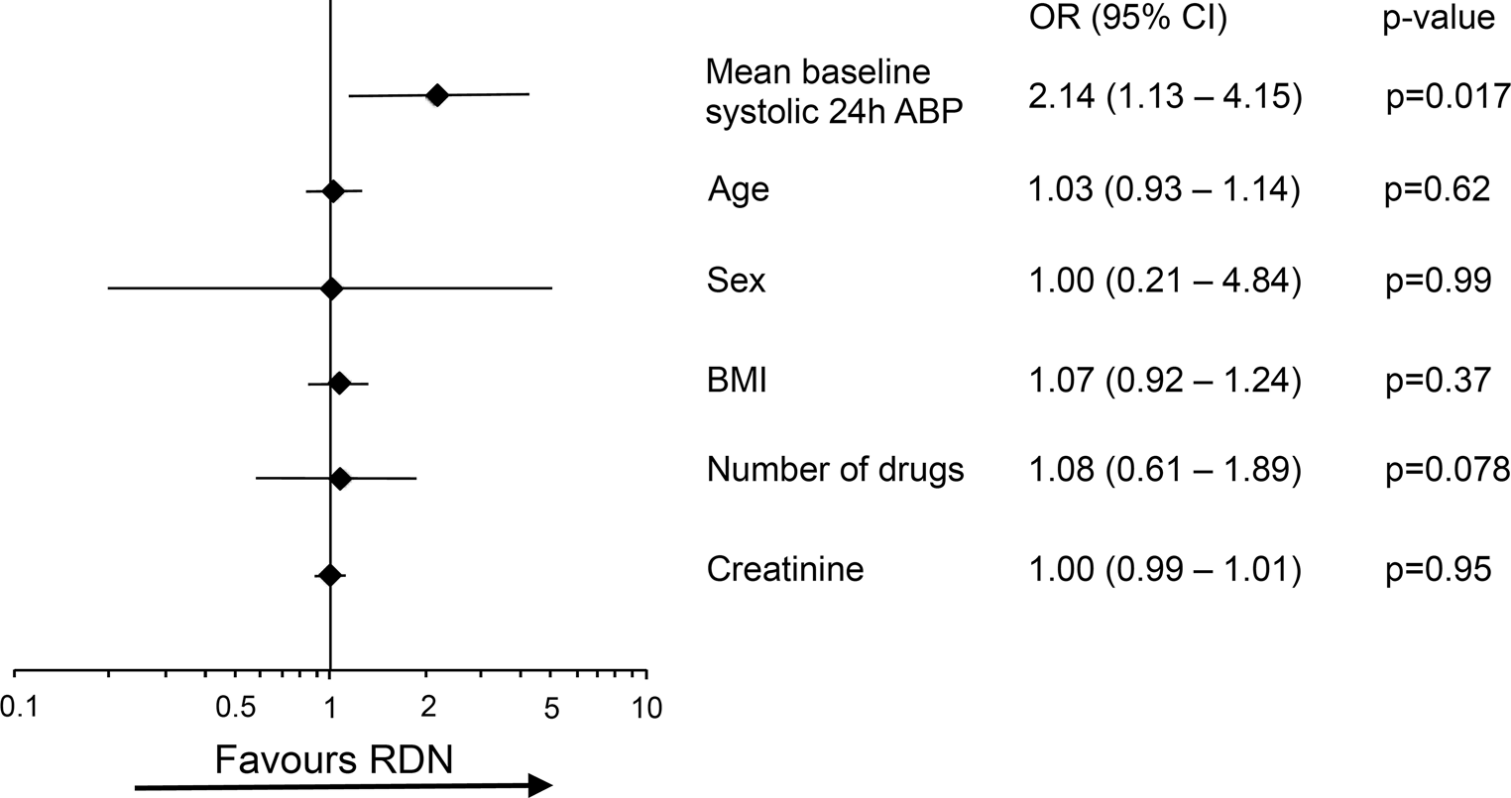
Multivariate logistic regression model for responders to renal denervation. Odds ratios (ORs) with 95 % confidence intervals (CIs) were calculated as an estimate of 24‑h ambulatory BP (ABP) reduction >10 mmHg at 12-month follow-up. Only the mean systolic 24‑h ABP at baseline was a predictor for responders of renal denervation therapy (OR 2.14, 95 % CI, 1.13–4.15; p = 0.017) whereas age, sex, body mass index (BMI), number of antihypertensive drugs at baseline and creatinine at baseline did not predict response to renal denervation

**Fig. 3:**
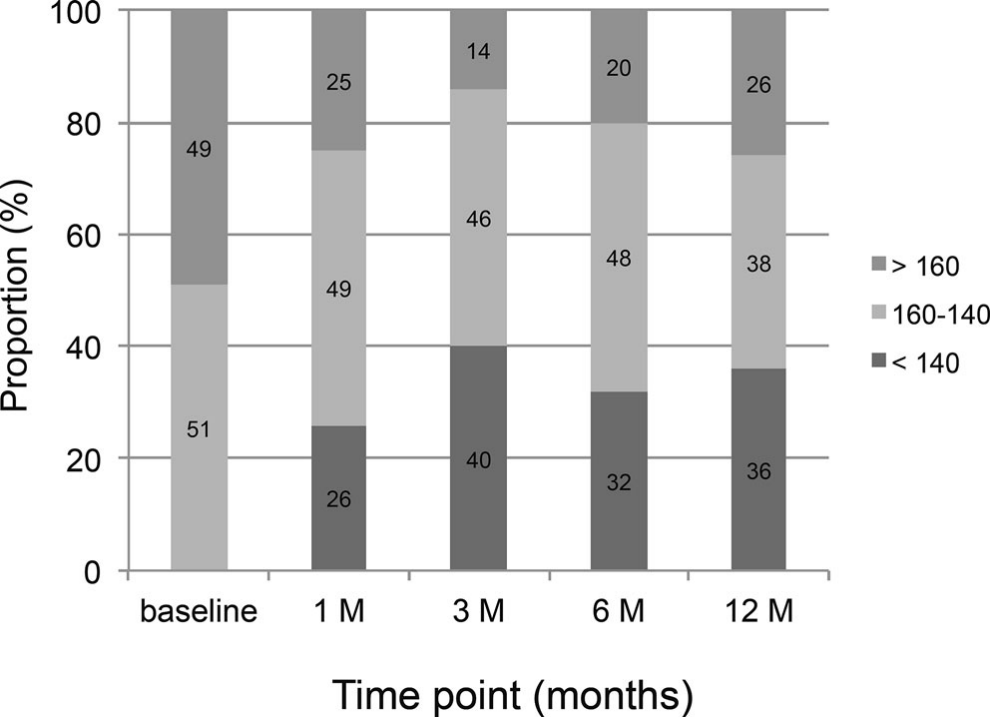
Distribution of systolic 24‑h ambulatory blood pressure at baseline and at 1/3/6/12-month follow-up

At 1/3/6/12 months we observed a reduction in mean systolic 24‑h ABP of −15 ± 19/−17 ± 17/−18 ± 18/−15 ± 18 mmHg (*n* = 55/53/57/50) and a mean diastolic 24‑h ABP of −6 ± 12/−9 ± 12/−10 ± 13/−7 ± 13 mmHg (Fig. 1). The absolute changes in mean ABP are shown in Table 2. Overall, there was a statistically significant BP reduction following RDN in mean systolic 24‑h ABP (χ^2^ (4) = 26.191, *p* < 0.001) as well as in mean diastolic 24‑h ABP (χ^2^ (4) = 20.771, *p* < 0.001). Wilcoxon signed-rank tests (with Bonferroni correction) showed significant BP changes in systolic 24‑h ABP (all p‑values < 0.0001) and diastolic 24‑h ABP at 1/3/6 months (all p‑values < 0.001) but not at 12 months (p = 0.013). Of the patients, 70 %/64 % showed a systolic 24‑h ABP reduction of ≥10 mmHg, and 77 %/70 % of ≥5 mmHg at 6/12 months of follow-up. Ten out of 70 patients could reduce and 4 out of 70 had to increase antihypertensive medication during follow-up. Logistic regression analysis with mean systolic 24‑h ABP reduction ≥10 mmHg at 12 months of follow-up as criterion revealed that only the baseline systolic 24‑h ABP was significant (OR 2.14, 95 % CI 1.13–4.15; p = 0.017) (Fig. 2). Fig. 3 shows the distribution of mean systolic 24‑h ABP at baseline, at 1/3/6/12 months of follow-up.

### Non-responders

Ten non-responder patients who did not show systolic 24‑h ABP reduction below 150 mmHg, and also did not show a reduction of systolic 24‑h ABP ≥10 mmHg at ≥3 months following RDN (time points ranging between 3–12 months), received second-line RDN and from this time point on were excluded from our analysis [13]. All other non-responders were analysed further.

### Safety

We did not observe any periprocedural complications. Ten of our patients missed the 12-month follow-up. All patients had at least one follow-up with duplex sonography of the renal artery. Two out of 75 treated patients (2.7 %) developed renal artery stenosis (>70 % lumen diameter) at 3‑ or 6‑month follow-up. In both patients stenting of the renal artery stenosis was performed without complications. However, one of these patients with newly diagnosed renal artery stenosis following RDN (with otherwise uncontrollable resistant hypertension) had previous stenting of the renal artery (in-stent stenosis). In all other patients (including the five patients who did not fulfil the inclusion criteria) duplex sonography could exclude significant renal artery stenosis. One of the patients with diabetic nephropathy developed worsening of renal function due to aggressive treatment with diuretics. Renal function recovered without further sequelae after reduction of the diuretics. In all other patients, we did not observe any renal or vascular complications during the 12 months of follow-up. The renal function of the patients remained stable (creatinine at baseline: 103 ± 48 µmol/l versus creatinine at 12-month follow-up: 100 ± 29 µmol/l). One patient died because of pulmonary embolism 9 months after the procedure (not related to the procedure).

## Discussion

To our knowledge, this is the study with the largest number of patients in whom RDN was performed with standard RF ablation catheter with to date the longest follow-up. In contrast to most of the currently available RDN studies, we exclusively used 24‑h ABP measurement for initial diagnosis and during follow-up. Using this strict setting, we observed excellent results in BP reduction with a high number of BP responders following the RDN procedure, with a very low rate of renal and cardiovascular complications. No periprocedural complications occurred.

Recently, the “real-world” data of the Global SYMPLICITY Registry, a prospective, open-label, multicentre registry with 998 patients, were published. In this registry, a reduction of −11.6 mmHg in systolic office BP and −6.6 mmHg in systolic 24‑h ABP was demonstrated at 6 months [14]. This BP reduction, although significant, was smaller than that observed in the HTN-1 and HTN-2 trials [5, 6]. Except that fewer patients were included in our study, the 24‑h ABP reduction observed was higher than described in the Global SYMPLICITY Registry [14]. Also the BP responder rate in our study at 6‑ and 12-month follow-up was higher (systolic 24‑h ABP reduction >10 mmHg at 6/12 months: 70 %/64 %; >5 mmHg: 77 %/70 %) in comparison with the Global SYMPLICITY Registry (systolic 24‑h ABP reduction at 6 months >8 mmHg: 48 %; >5 mmHg: 68 %) [14]. In 2015, two randomised controlled RDN trials (DENERHTN and Prague-15 study) were published [15, 16]. In the DENERHTN study 101 patients were randomised to receive either standardised stepped-care antihypertensive treatment alone (baseline mean systolic 24‑h ABP 156 mmHg) or in combination with RDN (baseline mean systolic 24‑h ABP 160 mmHg) [15]. In this study, the observed reduction in systolic 24‑h ABP following RDN with the Symplicity catheter (−15.8 mmHg at 6‑month follow-up) was comparable with our results (−18 mmHg). However, in the standardised hypertensive treatment group the 24‑h ABP reduction was −9.9 mmHg, amounting to an additional systolic BP reduction of −5.9 mmHg in the RDN group [15]. The responder rate in systolic 24‑h ABP was not described in the DENERHTN study [15]. In contrast, the Prague 15-study with 106 randomised patients found no significant differences in the 24‑h ABP between intensive pharmacological treatment and the RDN group. However, after 6 months, the number of antihypertensive drugs and the serum creatinine level was higher in the pharmacological treatment group [16]. Keeping in mind that the baseline patient characteristics were similar in the afore-mentioned studies, some important differences might explain the better BP response in our and in the DENERHTN study. First, the mean systolic 24‑h ABP was 165 mmHg in our and 159 mmHg in the DENERHTN study, whereas in the Global SYMPLICITY Registry the baseline mean systolic 24‑h ABP was 151 mmHg [14]. As described previously and confirmed in our and in the DENERHTN study, the baseline systolic office BP, or mean baseline systolic 24‑h ABP, are to date the only known independent predictors of BP response following RDN [15, 17, 18].

Second, 41 % of the operators in the Global Symplicity registry performed fewer than 15 RDN procedures with the Symplicity catheter. In contrast, in our single-centre study two experienced electrophysiologists performed all the procedures (>30 RDN procedures per operator). Therefore, the operators in our study might be more familiar with the handling of the RF ablation catheter. However, in the DENERHTN study with similar BP responses, the number of RDN procedures per operator was also below 10 cases [15].

Third, we used a 7‑F 4‑mm tip standard RF ablation catheter that might provide a higher contact pressure, higher tip stability and greater torque control during ablation in comparison with the first-generation Simplicity catheter. Animal studies conducted by our group found that the RF ablation for RDN with a standard RF ablation catheter can be performed without harmful sequelae. Further, we observed an attenuation of neurofilament expression in the renal artery as a surrogate marker of effective RDN [19]. The performance of these standard RF ablation catheters is non-traumatic as shown in our study (no acute complications in the renal artery), and also during RF ablation of the coronary venous system and the aortic sinus for ablation of ventricular arrhythmias [20]. In comparison to the results of the DENERHTN study and the Global Symplicity registry, RDN using a 7‑F 4‑mm tip standard RF ablation catheter seems to be at least as effective as RDN with the first-generation Simplicity catheter.

According to our data and to data in the literature, there is a risk of the development of renal artery stenosis following RDN. We observed two new renal artery stenoses (2.7 %) in our patient sample, detected by duplex sonography of the renal artery. However, in one of these patients (with otherwise uncontrollable resistant hypertension) the stenosis was an in-stent-restenosis after renal artery stenting that was performed years ago. In contrast, renal artery stenoses were reported to range between 0.3–1.9 % in the Symplicity HTN-1, -2 and -3 trials [8, 21]. No renal artery stenosis was reported in the Global SYMPLICITY Registry and the DENERHTN study. However, one could speculate that renal artery stenosis might be overlooked in the Global SYMPLICITY Registry since, as in our study, “there was no mandatory renal artery imaging follow-up” [14] and, especially in overweight patients, duplex sonography is considered an inferior imaging modality to magnetic resonance angiography or angio-CT. In the EnligHTN 1 study (*n* = 46) renal artery stenosis occurred in 4.3 % of the treated patients [22]. In all these cases this resolved after treatment with renal artery stenting, without further complications.

The effect of RDN between the office BP and the 24‑h ABP measurements differed across the known RDN studies, reviewed by Verloop et al. and Bunte [23, 24]. One reason for this might be that the 24‑h ABP was not the primary study endpoint (HTN-1 and 2) [7, 8] or the required level for study inclusion was rather low (systolic 24‑h ABP >135 mmHg in HTN-3 and Global SYMPLICITY Registry) [9, 14]. However, the 24‑h ABP measurement performed in the patient’s own environment provides more objective results than the office-based BP [25]. In our opinion, 24‑h ABP measurement with BP values that are beyond 135/90 mmHg as an inclusion criterion should be used for further RDN trials since a systolic 24‑h ABP of 135 mmHg might be too close to the normal range. The weaker criterion of office-based BP should only be used as a sub-criterion.

There are some limitations to our study worth noting. One general concern raised by the negative results of the first randomised sham-controlled study (HTN-3) [9] is whether observation studies can still be deemed useful for evaluating the efficacy of the percutaneous RDN procedure. As a matter of fact, randomised controlled trials are the gold standard in testing efficacy of medical interventions [26]. Observational studies can be an alternative, however, when randomised controlled trials are difficult to perform [27]. As a practical alternative to doing nothing, well-designed prospective observational studies might be used to obtain more information on treatment effect and long-term outcome [28]. The HTN-3 trial had some methodological deficiencies, mentioned above, and there is evidence from other randomised controlled trials and also from many observational studies that RDN is effective and safe [6, 8, 14, 17, 22]. The sample size in our study with 70 patients analysed is rather small. As we used 24‑h ABP measurement consequently, it is to date the largest number and longest follow-up of patients in whom RDN was performed by using a standard RF catheter. Because of the observational design of our study, we cannot rule out that the Hawthorne effect (a phenomenon that individuals modify their behaviour if they are aware they are being observed) [29] might contribute to the BP reduction observed. Another problem of observational studies is the regression to the mean (Extreme outliers on the first measurement tend to be less extreme and closer to the mean on the second measurement. It can make natural variation in repeated data assessments look like a real change.) [30]. However, all of the patients included in our study had a long history of resistant hypertension with multiple previous clinical consultations and examinations, BP measurements, intensive review, and adaption of medication without significant effects on 24‑h ABP. Furthermore, the patients had to be on a stable antihypertensive treatment for at least 4 weeks before inclusion in our study.

In conclusion, as shown during long-term follow-up our results support the hypothesis that RDN with a standard RF ablation catheter can be used safely to substantially reduce mean 24‑h ABP in resistant hypertension, with an acceptable risk for the development of renal artery stenosis. Thus, our findings support and extend the results of previous studies that RDN is effective in BP reduction. In our study, only the mean systolic 24‑h ABP at baseline was a predictor for responders and should therefore be used as the inclusion criterion for further RDN studies. However, for a final judgement of the efficacy of RDN, randomised trials with long follow-ups are needed.
